# ABC1K10a, an atypical kinase, functions in plant salt stress tolerance

**DOI:** 10.1186/s12870-020-02467-4

**Published:** 2020-06-10

**Authors:** Xiaohui Qin, Zhikun Duan, Yuan Zheng, Wen-Cheng Liu, Siyi Guo, José Ramón Botella, Chun-Peng Song

**Affiliations:** 1 State Key Laboratory of Crop Stress Adaptation and Improvement, School of Life Sciences, Henan University, Kaifeng, China; 2 State Key Laboratory of Cotton Biology, School of Life Sciences, Henan University, Kaifeng, China; 3 School of Agriculture and Food Sciences, University of Queensland, Brisbane, Australia

**Keywords:** ABC1K, ABC1K10a, Salt stress, Reactive oxygen species, Mitochondria

## Abstract

**Background:**

ABC1K (Activity of BC1 complex Kinase) is an evolutionarily primitive atypical kinase family widely distributed among prokaryotes and eukaryotes. The ABC1K protein kinases in *Arabidopsis* are predicted to localize either to the mitochondria or chloroplasts, in which plastid-located ABC1K proteins are involved in the response against photo-oxidative stress and cadmium-induced oxidative stress.

**Results:**

Here, we report that the mitochondria-localized ABC1K10a functions in plant salt stress tolerance by regulating reactive oxygen species (ROS). Our results show that the ABC1K10a expression is induced by salt stress, and the mutations in this gene result in overaccumulation of ROS and hypersensitivity to salt stress. Exogenous application of the ROS-scavenger GSH significantly represses ROS accumulation and rescues the salt hypersensitive phenotype of *abc1k10a*. ROS overaccumulation in *abc1k10a* mutants under salt stress is likely due to the defect in mitochondria electron transport chain. Furthermore, defects of several other mitochondria-localized *ABC1K* genes also result in salt hypersensitivity.

**Conclusions:**

Taken together, our results reveal that the mitochondria-located ABC1K10a regulates mitochondrial ROS production and is a positive regulator of salt tolerance in Arabidopsis.

## Background

High salinity in soil is a world-wide environmental condition that inhibits the growth and reduces the yields of crops [1, 2]. High concentrations of sodium (Na^+^), a major ion in saline soils, result in osmotic stress, Na^+^ toxicity and oxidative stress, and thus cause salt damages to plants [3]. The salt overly sensitive (SOS) pathway is a core mechanism for plants to diminish cytosolic accumulation of Na^+^ and maintain cellular K^+^/Na^+^ homeostasis [4]. In the SOS pathway, salt stress elicits the activity of calcium binding proteins SOS3 and SOS3-LIKE CALCIUM BINDING PROTEIN 8 (SCaBP8), which activate the protein kinase SOS2, resulting in the phosphorylation and activation of the Na^+^/H^+^ antiporter SOS1 and thus enhanced Na^+^ efflux from cytosol [5]. The SOS pathway is regulated by several proteins in response to salt stress. Under normal growth conditions, the SOS2 kinase activity is repressed by the PROTEIN KINASE SOS2-LIKE 5 (PKS5), 14–3-3 proteins and GIGANTEA (GI), leading to a decreased Na^+^/H^+^ antiporter activity of SOS1 [6, 7]. Salt stress promotes the interaction between PKS5 and 14–3-3 s, which releases the inhibition of SOS2, resulting in the activation of the SOS pathway to regulate Na^+^ homeostasis [6]. A putative calcium-permeable transporter AtANN4 is phosphorylated and repressed by the SOS2-SCaBP8 complex, which fine-tunes the calcium signal and modulates the SOS pathway in response to salt stress [8].

Salt stress induces the overaccumulation of reactive oxygen species (ROS) such as hydrogen peroxide (H_2_O_2_), singlet oxygen (^1^O_2_), hydroxyl radical (•OH), and superoxide anion radical (O_2_˙ˉ) [3, 9]. Under salt stress, ROS can be generated from multiple cellular locations including chloroplasts, mitochondria, peroxisomes and plasma membrane NADPH oxidases [10]. The mutation of rice gene *WSL12* that encodes chloroplast nucleoside diphosphate kinase 2 (NDPK2), results in enhanced ROS accumulation and sensitivity to salt stress, suggesting that chloroplast is a source of damaging ROS elicited by salt stress [11]. High salinity also impairs mitochondria electron transfer rates between different respiratory chain complexes and causes ubiquinone (UQ) over-reduction, and the excess electrons are transferred to molecular oxygen or nitrate, giving rise to O_2_˙ˉ or nitric oxide (NO) [12]. The mitochondrial protein AtMT2b (a type 2 metallothionein) is involved in plant salt stress response by interacting with the voltage-dependent anion channel 3 (AtVDAC3) to regulate ROS homeostasis and mitochondrial membrane potential (MMP) [13].

Aerobic organisms have evolved both non-enzymatic and enzymatic antioxidant defense mechanisms to protect plants against oxidative stress [14]. Non-enzymatic antioxidants include vitamin C, vitamin E, alkaloids, carotenoids, tripeptide glutathione (GSH) and flavonoids, while antioxidant enzymes such as superoxide dismutases (SODs), catalase (CAT), ascorbate peroxidase (APX) and glutathione peroxidase (GPX) can effectively detoxify cellular O_2_˙ˉ and H_2_O_2_ [10, 15]. Numerous studies have reported that overexpression of SODs (Cu/Zn-SOD, Mn-SOD, or Fe-SOD) enhances salt tolerance of the transgenic plants [15]. The transgenic rice overexpressing the *Escherichia coli* CAT encoded gene, *katE*, shows improved tolerance to salt stress [16]. Furthermore, as one of the most important metabolites, GSH functions in mitigating oxidative stress, and cellular GSH concentrations directly affect plant stress tolerance [15]. Therefore, maintaining cellular ROS homeostasis under stress conditions is important in plant stress response.

The bc1 complex kinases (ABC1Ks) are atypical kinases which lack many of the characteristics of eukaryotic protein kinases. The founder member of ABC1Ks is ABC1/COQ8 (now called ScCOQ8) from *Saccharomyces cerevisiae*, which functions to maintain the activity of the mitochondrial bc1 complex by regulating UQ synthesis [17]. As homologues of ScCOQ8, UbiB in *Escherichia coli*, aarF in *Providencia stuartii* and CABC1 in *Homo sapiens* also function in UQ biosynthesis [18, 19]. The *Arabidopsis* ABC1K family contains 17 members, eight of which (AtABC1K1–8) belong to the photosynthetic-specific clade, six (AtABC1K11–15) belong to the mitochondrial clade, and three (AtABC1K9, AtABC1K10a and AtABC1K10b) are ancestral clade members [20, 21]. AtABC1K8 (AtOSA1) is the first identified member of this family localized in chloroplasts and participates in mitigating cadmium-induced oxidative stress [22]. ABC1K1 and its homolog ABC1K3 phosphorylate VTE1, a major limiting factor in tocopherol synthesis, and participate in plant response to photo-oxidative stress [23–26]. The chloroplastic ABC1Ks have been extensively studied, but the role of mitochondrial ABC1Ks remains unclear.

In this study, we demonstrate that the mitochondrial ABC1K10a plays an important role in salt stress tolerance. The *ABC1K10a* knock-out mutants accumulate excessive H_2_O_2_ and O_2_˙ˉ and are hypersensitive to salt stress. Inhibition of ROS synthesis alleviated the salt stress hypersensitivity of *abc1k10a* mutants. Further analysis showed that the excessive ROS accumulation in *abc1k10a* under salt stress is likely to be resulted from irregularities of the respiratory complex in mitochondria. Other mitochondrial ABC1K members are also involved in the response to salt stress by regulating ROS accumulation, indicating the important roles of ABC1K family in salt stress response. Taken together, the outcome of our results sheds new light on the positive role of the mitochondria-located ABC1K10a in the regulation of salt tolerance in Arabidopsis.

## Results

### *Arabidopsis* mitochondria-*abc1k* mutants accumulate higher ROS under salt and osmotic stresses

**Fig. 1:**
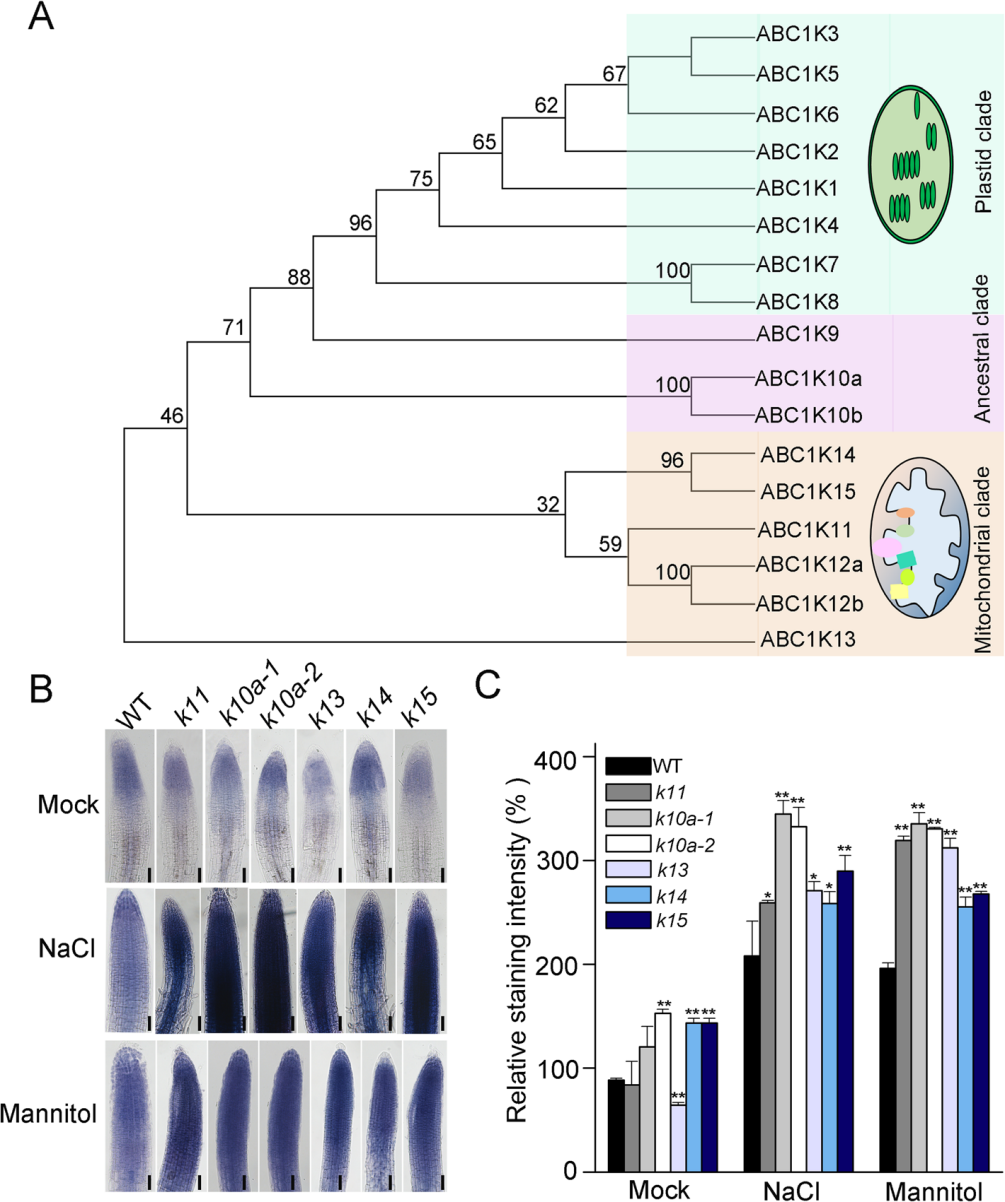
The *abc1k* mutants accumulate more ROS than the wild type under salt and osmotic stresses. (**a**) Phylogenetic tree of ABC1K proteins from *Arabidopsis*. The tree was constructed by comparing the amino acid sequences of all 17 ABC1K proteins encoded in the *Arabidopsis thaliana* genome. Three different clades were highlighted with different colors (green indicates the plastids clade; purple indicates the ancestral clade, and light pink indicates the mitochondria clade) according to previous reports [20, 21]. (**b**) NBT staining of the roots of wild type, *abc1k11*, *abc1k10a*, *abc1k13*, *abc1k14* and *abc1k15* mutant roots treated with 200 mM NaCl or 300 mM mannitol. Bars = 400 μm. *k*, *abc1k.* (**c**) Quantification of NBT staining intensity. Asterisks indicate statistically significant difference with wild type plants in each treatment. Error bars indicate ± SD (*n* = 15) (**P < 0.01, *P < 0.05, *t*-test)

The members of *Arabidopsis* ABC1K family can be divided into three clades according to their evolutionary origins and subcellular localizations: chloroplast clade, mitochondrial clade and ancestral clade (Fig. 1a). Previous studies demonstrated that ABC1K proteins located in chloroplast are involved in oxidative stress response [22, 24]. To determine whether the ancestral clade and mitochondrial clade of ABC1Ks are also involved in ROS metabolism, we detected the ROS levels in several *abc1k* mutants under NaCl or mannitol treatment. ROS levels in most *abc1k* mutants increased significantly under both treatments, suggesting a role for these proteins in ROS detoxification under abiotic stress conditions. The results revealed the highest ROS accumulation in two independent *abc1k10a* mutants (*k10a-1* and *k10a-2*) and this gene was chosen for further characterization (Fig. 1b, c).

### The expression pattern of *ABC1K10a (K10a)* gene

**Fig. 2:**
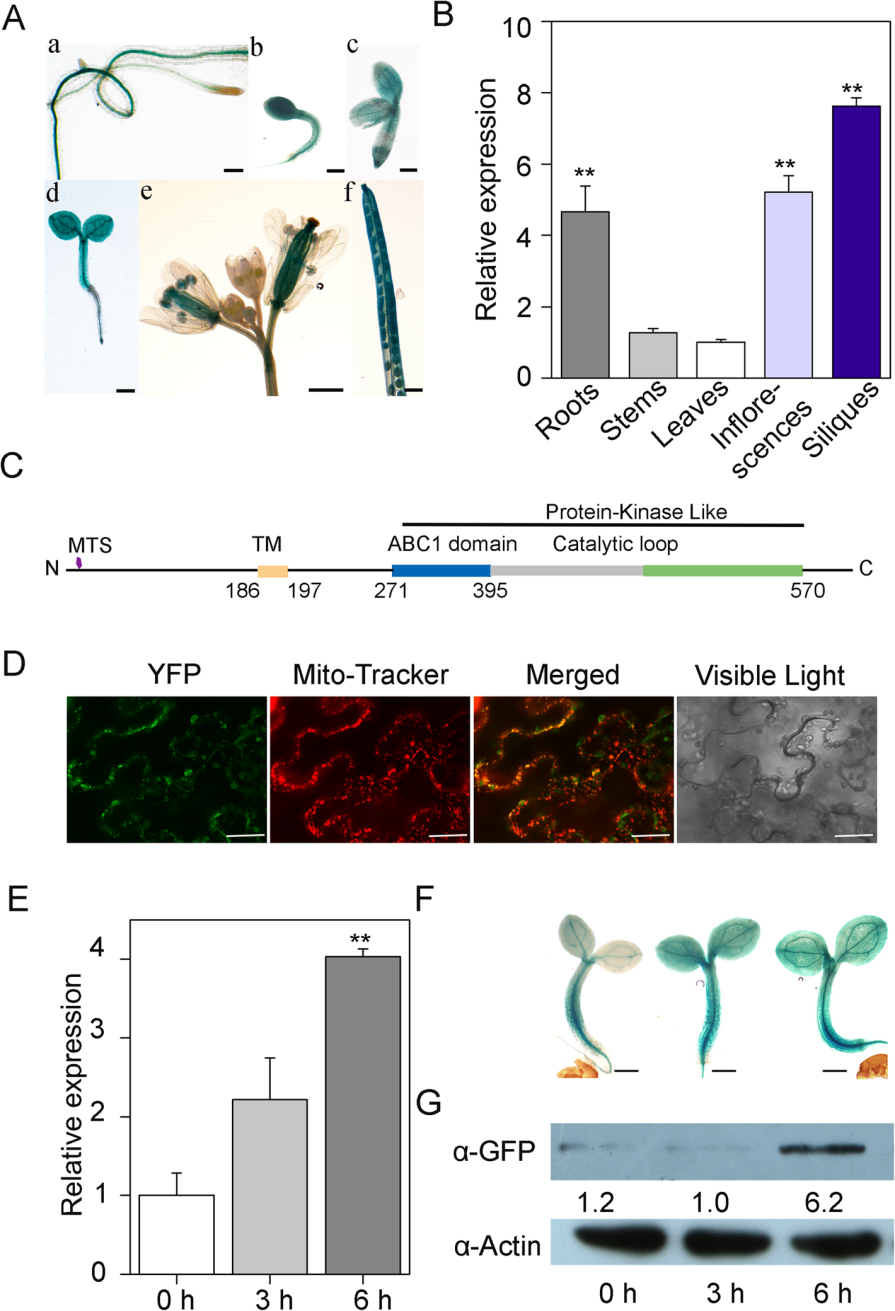
Molecular characterization of *ABC1K10a*. (**a**) GUS staining of transgenic Arabidopsis lines carrying a *proK10a::GUS* construct. b, e and f, bars = 2 mm. a, c and d, bars = 500 μm. (**b**) Expression of *ABC1K10a* in different tissues detected by qRT-PCR. *ACTIN2/8* was used as a control. Asterisks indicate statistically significant difference between leaves and other tissues (**P < 0.01, *t*-test). (**c**) Schematic protein structure of ABC1K10a. MTS, mitochondrial targeting sequence; TM, transmembrane domain. (**d**) Subcellular localization of ABC1K10a (green fluorescence) and Mito-Tracker (red fluorescence) in *Nicotiana benthamiana* leaves. Bars = 50 μm. (**e, f &g**) Expression of ABC1K10a is induced by NaCl treatment. (E) Relative transcript levels of *ABC1K10a* (qRT-PCR) under salt stress were obtained by comparing treated and untreated samples. Error bars indicate ± SD (*n* = 3). Asterisks indicate statistically significant differences between treated and untreated samples at the different treatment times (**P < 0.01, *t*-test). (**f**) For GUS staining, 5 day-old transgenic seedlings expressing *ABC1K10apro::GUS* were treated with 200 mM NaCl for 0, 3 or 6 h. Bars = 200 μm. (**g**) To extract the total proteins, 5 day-old transgenic seedlings expressing *ABC1K10a pro::ABC1K10a-GFP* were treated with 200 mM NaCl for 0, 3 or 6 h. Protein extracts were analyzed by western blotting with α-GFP antibody (**g**). Actin was used as a control

The expression patterns of *ABC1K10a* (named *K10a* herewith) were first analyzed by using *proK10a::GUS* transgenic plants. GUS staining showed that *K10a* was expressed in various tissues, with relatively higher expression in siliques and cotyledons (Fig. 2a). These results were further confirmed by qRT-PCR analysis, which showed the strongest expression levels in roots, flowers and siliques (Fig. 2b). Bioinformatics analysis indicated a putative mitochondrial signal peptide at the N-terminus (Fig. 2c), suggesting a mitochondrial localization of K10a. To verify this, the K10a-YFP fusion proteins were expressed in *Nicotiana benthamiana* leaves, and the fluorescence of K10a-YFP was exclusively detected in the mitochondria, overlapping with the mitochondria marker Mito-tracker red (Fig. 2d). This result indicates that the K10a is indeed a mitochondrial protein.

To obtain support for a putative role for the mitochondrial K10a protein in abiotic stress we determined the expression patterns under salt stress treatment. The results of qRT-PCR and GUS staining showed that the transcript levels of *K10a* were up-regulated by NaCl treatment (Fig. 2e, f). Furthermore, analysis of transgenic lines expressing a K10a-GFP fusion protein under the control of the *K10a* native promoter (*proK10a::K10a-GFP*) confirmed that NaCl treatment induces a marked increase in K10a protein levels (Fig. 2g; Additional file 2: S2). These results indicate that *K10a* is a salt inducible gene and may play a role in salt stress response.

### The *abc1k10a* mutants are hypersensitive to salt stress

**Fig. 3:**
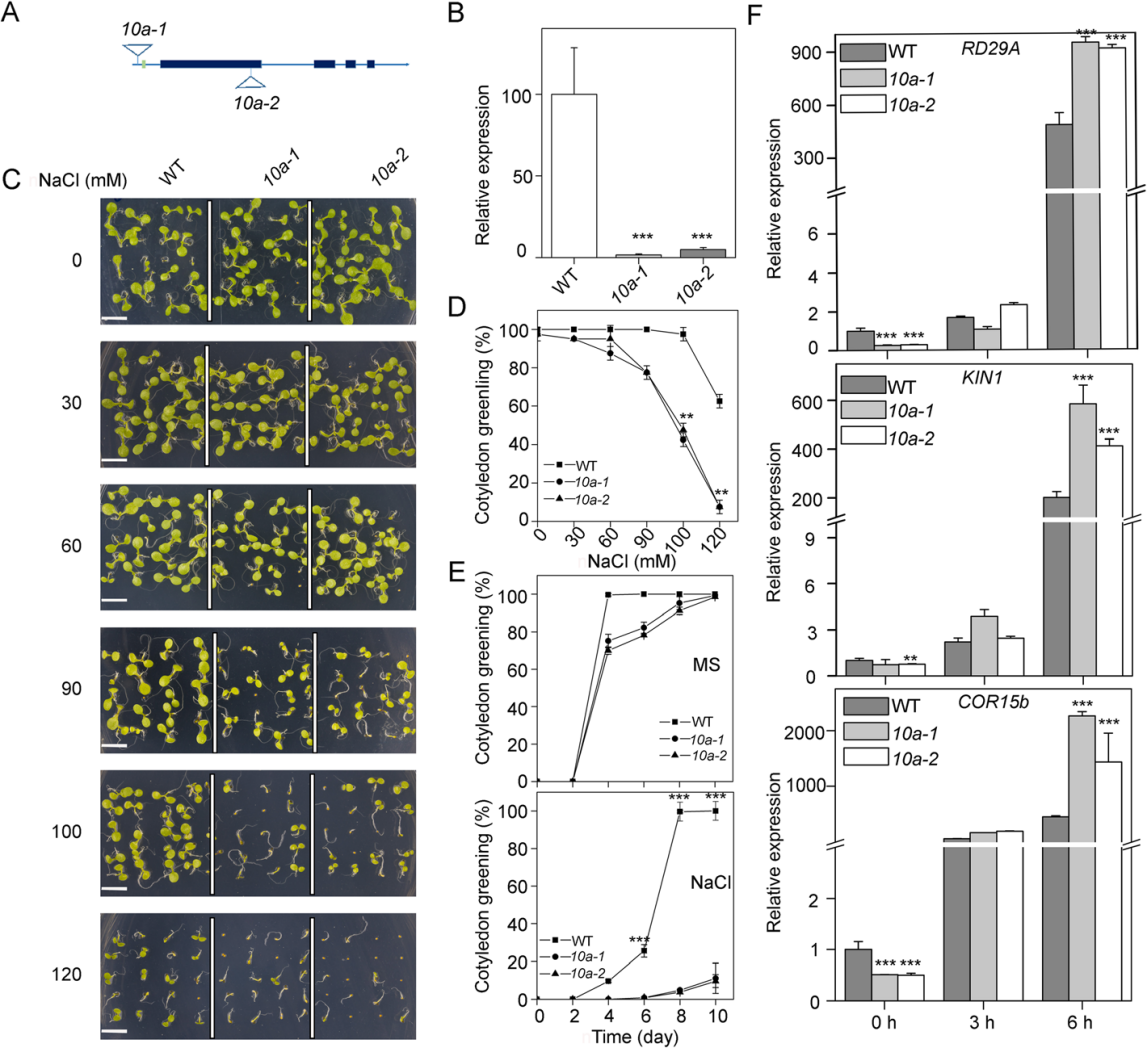
The *abc1k10a* mutants show increased sensitivity to salt stress.(**a**) Schematic diagram of the *ABC1K10a* gene. The filled boxes represent exons, while the lines between the boxes represent introns. Two T-DNA insertions are also indicated. (**b**) Transcript abundance of *ABC1K10a* in the wild type and *abc1k10a* mutants. Total RNAs isolated from 5 day-old wild type and *10a* mutant seedlings were used for real-time qRT-PCR. Three biological replicates were conducted (means ± SD, *n* = 3). *10a*, *abc1k10a*. Asterisks indicate statistical difference between *10a* mutants and wild type (***P < 0.001, *t-*test). (**c**) Salt sensitivity of wild type and *10a* mutants. Wild type, *10a-1* and *10a-2* mutants seeds were germinated and grown for 8 days on MS medium containing different concentrations of NaCl. Bar = 500 μm. (**d**) Cotyledon greening of seedlings grown on MS medium containing different concentrations of NaCl was scored after 10 days and represent averages of 100 seedlings from at least four independent experiments. (means ± SD; *n* = 100). Asterisks indicate statistically significant differences (**P < 0.01, *t-test*) between *10a* mutants and wild type. (**e**) Cotyledon greening rates in wild type and *10a* mutants in response to NaCl treatment. Wild type, *10a-1* and *10a-2* seeds were sown on MS medium or MS medium with 100 mM NaCl. Cotyledon greening rates were calculated at the indicated days. Error bars indicate ± SD (*n* = 100). Asterisks indicate statistically significant differences (***P < 0.001, *t-*test) between the *10a* mutants and wild type. (F) The transcript levels of *RD29A*, *KIN1* and *COR15b* in wild-type, *10a-1* and *10a-2* mutants at different times after treatment with NaCl. 5-day-old seedlings were treated without or with 200 mM NaCl at the indicated times, and transcript levels were analyzed by qRT-PCR. Error bars indicate ± SD (*n* = 3). Asterisks indicate statistically significant differences between the *10a* mutants and wild type (**P < 0.01, ***P < 0.001, t-test)

To study the putative role of K10a during salt stress response, we characterized the behavior of two independent T-DNA mutants (*10a-1* and *10a-2*) showing negligible *K10a* expression (Fig. 3a, b). Under normal growth conditions, the *10a* mutants showed similar growth with the wild type. Under NaCl treatment, however, the *10a* mutants displayed more severely inhibited growth than the wild type (Fig. 3c, d, e). Effects on cotyledon greening were clearly observed on *10a* mutants at lower NaCl concentrations than WT (Fig. 3d). After growing for 10 days on MS medium containing 100 mM NaCl, all the wild type seedlings had green cotyledons, while only 11% of the *10a-1* and 10% of the *10a-2* mutants had green cotyledons (Fig. 3e). Expression analysis of the salt-responsive marker genes of *Responsive to Desiccation 29A* (*RD29A*) [27], *Kinase 1* (*KIN1*) [27] and *Cold Regulated 15b* (*COR15b*) [28] showed significantly higher expression in the *10a* mutants than in wild type after salt treatment for 6 h (Fig. 3f). Collectively, these results indicate that K10a is important in plant salt stress response and tolerance.

### *Arabidopsis* ABC1K10a positively regulates salt stress tolerance

**Fig. 4:**
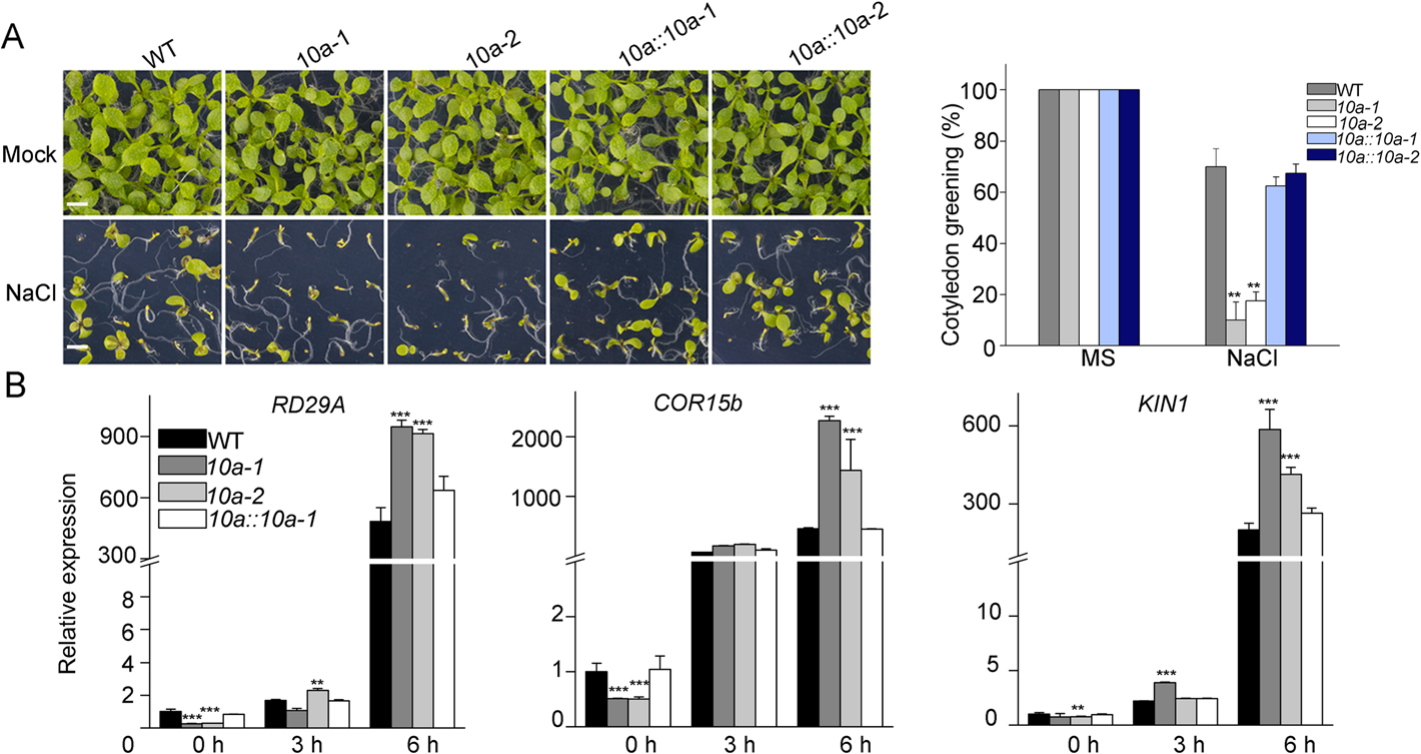
Complementation of *10a* mutants restores salt sensitivity to wild type levels. (**a**) Arabidopsis seedlings grown in the absence or presence of 100 mM NaCl for 12 days. Complementation lines *10a::10a-1* and *10a::10a-2* were obtained by introducing a *ProABC1K10a::ABC1K10a-GFP* construct into the *10a* mutant background (Bar = 500 μm). Asterisks indicate statistically significant difference compared to the wild type in the same treatment (**P < 0.01, *t*-test). (**b**) Relative expression levels of *RD29A*, *KIN1* and *COR15b* in wild-type, *10a-1*, *10a-2* and *10a::10a-2-GFP* seedlings analyzed by qRT-PCR. Asterisks indicate statistically significant differences with WT (**P < 0.01, t-test)

To further confirm that the salt hyper-sensitive phenotype of the *10a* mutants is caused by the defect of *K10a*, two independent complementation transgenic lines were generated by introducing the wild type *K10a* gene driven by the native *K10a* promoter into the *10a-2* mutant. The expression of *K10a* in the *10a-2* mutant fully restored the salt hypersensitive phenotype of the *10a-2* mutant to wild type levels, further supporting that *K10a* is required for salt tolerance in *Arabidopsis* (Fig. 4a). Consistently, the expression levels of *RD29A*, *KIN1* and *COR15b* in the complementation lines were also restored to the levels of wild type in the presence or absence of salt stress (Fig. 4b). In addition, we generated transgenic lines overexpressing *K10a-Myc* under the control of *35S* promoter (*K10a-OE*), and the *K10a-OE* lines displayed enhanced salt tolerance when compared with the wild type (Additional file 1: S1). Overall, our results indicate that K10a positively regulates plant salt tolerance.

### ROS overaccumulation results in the salt hypersensitivity of *10a* mutants

**Fig. 5:**
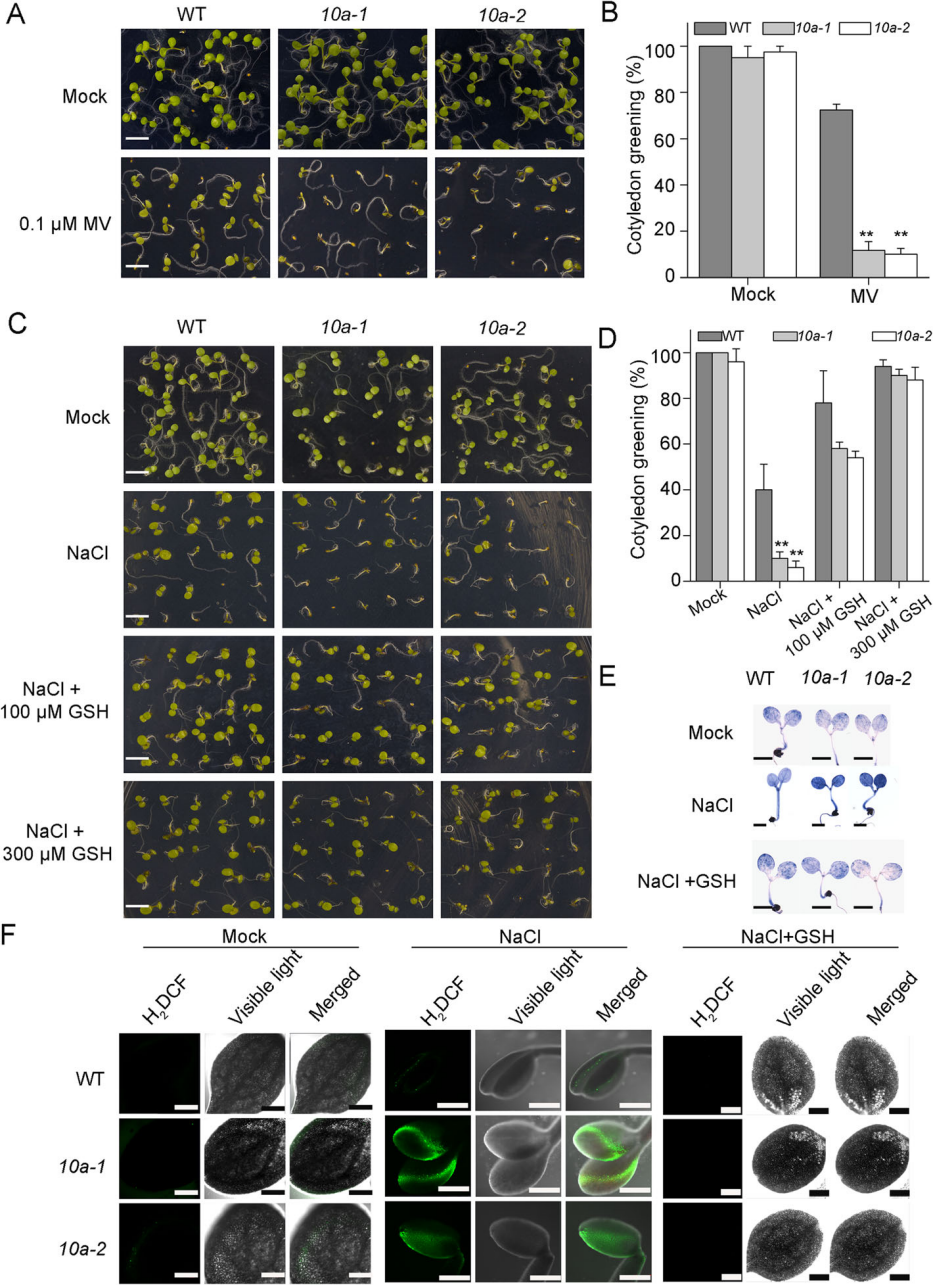
ROS scavenging can reverse the hypersensitivity to salt stress of *10a* mutants. (**a**) Phenotypes of wild type and *10a* mutants in response to MV treatment. Seeds were sown on MS medium with or without the addition of 0.1 μM MV and photographed after 10 days (**a**) (Bar = 500 μm). Rates of cotyledon greening were calculated in (**b**) (means ± SD, *n* = 100). Asterisks indicate statistically significant differences with the corresponding wild type (**P < 0.01, *t*-test). (**c**) Alleviation of salt stress symptoms by GSH treatment in *10a* mutants. Seedlings grown on the indicated media for ten days were photographed and the rates of cotyledon greening were calculated (**d**). (Bar = 500 μm. means ± SD, *n* = 100). Asterisks indicate statistically significant differences with the corresponding wild type (**P < 0.01, *t-test*). (**e** and **f**) NBT staining (**e**) and H_2_DCF-DA staining (**f**) of the cotyledons of wild type and *10a* mutants after NaCl treatment or supplemented with 100 μM GSH. Bars = 2 mm (**e**), bars = 300 μm (**f**)

Our results showed that NaCl treatment resulted in higher ROS accumulation in the *10a* mutants, therefore we wondered whether the increased ROS is the main reason of the observed salt hypersensitive phenotypes (Fig. 1b, c). Methyl viologen (MV) induces the production of ROS in plant chloroplasts and mitochondria [29]. When plants were grown in MS media supplemented with 0.1 μM ROS-generating chemical methyl viologen MV, the *10a* mutants displayed hypersensitivity to MV, similar phenotypes to those observed under salt stress (Fig. 3c; Fig. 5a). In the presence of MV, the *10a* mutants had only 11% green cotyledons, compared with 70% green cotyledons in the wild type (Fig. 5b). These results suggest that the salt hypersensitive phenotype of *10a* mutants may be resulted from ROS overaccumulation.

We used the ROS-scavenger GSH to determine the involvement of ROS in the salt stress response of *10a* mutants. After germination in MS medium containing 100 mM NaCl for six days, 40% of wild type seedlings grew green cotyledons, while only 10% of the *10a-1* and 6% of the *10a-2* mutants grew green cotyledons (Fig. 5c, d). However, the addition of 300 μM GSH to the medium neutralized the effect of salt with WT, *10a-1* and *10a-2* seedling cotyledon greening reaching 94, 90 and 88%, respectively, indicating that the growth inhibition by NaCl treatment was resulted from ROS accumulation and the salt hypersensitivity of the *10a* mutants was due to overaccumulation of ROS (Fig. 5c, d). ROS were detected by NBT and 2′7’-dichlorofluorescin diacetate (H_2_DCF-DA) staining, which indicate O_2_˙ˉ and H_2_O_2_, respectively [30]. Under normal growth conditions, no differences were observed between wild type and *10a* mutants. However, NaCl treatment produced a significantly stronger staining in the *10a* mutants compared to wild type, while GSH reduced the excess ROS accumulation in all genotypes (Fig. 5e, f). These results further support that ROS overaccumulation under salt stress accounts for the salt hypersensitivity of *10a* mutants.

### The *10a* mutations cause dysfunction of mitochondria under salt stress

**Fig. 6:**
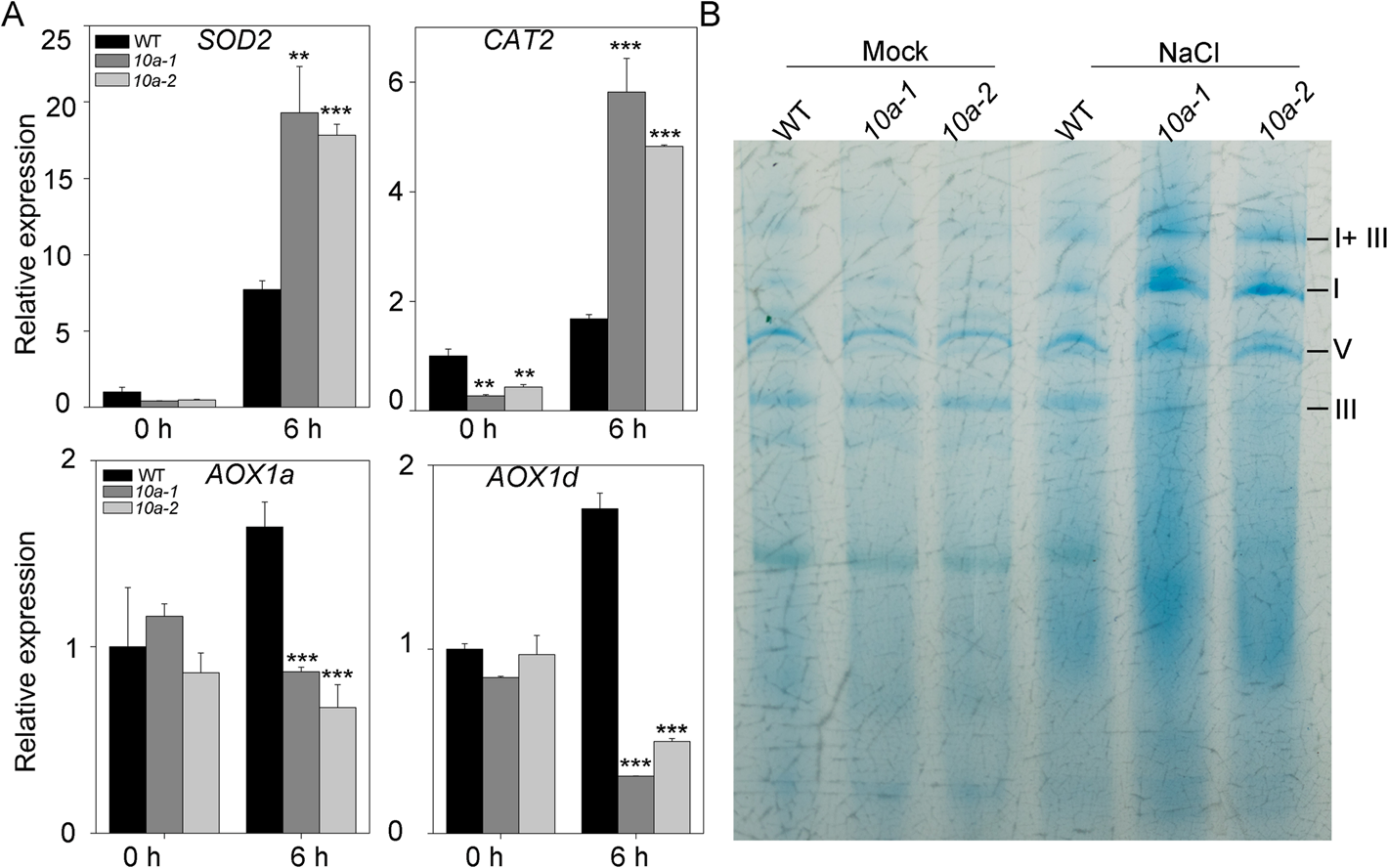
Dysfunction of the mitochondria in *abc1k10a* mutants under salt stress. (**a**) Expression of the oxidative stress related genes. Expression of *SOD2*, *CAT2*, *AOX1a* and *AOX1d* in plants treated with 200 mM NaCl was determined by qRT-PCR. Error bars indicate ± SD (*n* = 3). Asterisks indicate statistically significant differences with the corresponding wild type (**P < 0.01 and ***P < 0.001, *t*-test). (**b**) The abundance of the mitochondrial complexes in wild type and the *10a* mutants. Crude mitochondrial membranes were extracted from 5-day-old seedlings grown on MS medium or MS medium supplemented 100 mM NaCl. The mitochondrial complexes were separated by Blue Native-PAGE and stained with coomassie brilliant blue

Mitochondria are important sources of ROS production, especially under various abiotic stresses which restrain ETC activity, disturb electron transfer and promote ROS production [10]. Under salt stress, the classic cytochrome c oxidase (COX) pathway is inhibited, and the alternative oxidase (AOX) pathway plays an important role transferring electrons from ubiquinone to oxygen and limiting ROS production [31, 32]. To determine whether the high level of ROS induced by salt stress in *10a* mutants resulted from mitochondrial dysfunction, we analyzed the expression of *AOX* genes and two antioxidant genes. Under NaCl treatment, the expression of *SOD2* and *CAT2* was significantly increased in the *10a* mutants compared to the wild type, suggesting that the*10a* mutants suffered more severe oxidative stress than the WT (Fig. 6a). However, lower expression of *AOX* gene in the *10a* mutants than in the wild type implied that AOX pathway was severely restrained, resulting in ROS over-production (Fig. 6a).

We performed Blue Native (BN)-PAGE analyses to determine the assembly of the complexes in the mitochondrial respiratory chain. Similar levels of the mitochondrial complexes were detected between wild type and *10a* mutants under normal growth conditions. However, the complex III levels were more severely inhibited in the *10a* mutants under NaCl treatment when compared with the WT (Fig. 6b; Additional file 3: S3). Since the mitochondrial complex I and III are the key sites resulting in ROS production [12], the abnormality of the complex III assembly under salt stress may account for the overaccumulation of ROS in the *10a* mutants. These results also suggest that K10a may play a role in maintaining the integrity and stability of the complex III under salt stress in plants.

### Defects in other *ABC1K* genes also result in salt hypersensitivity

**Fig. 7:**
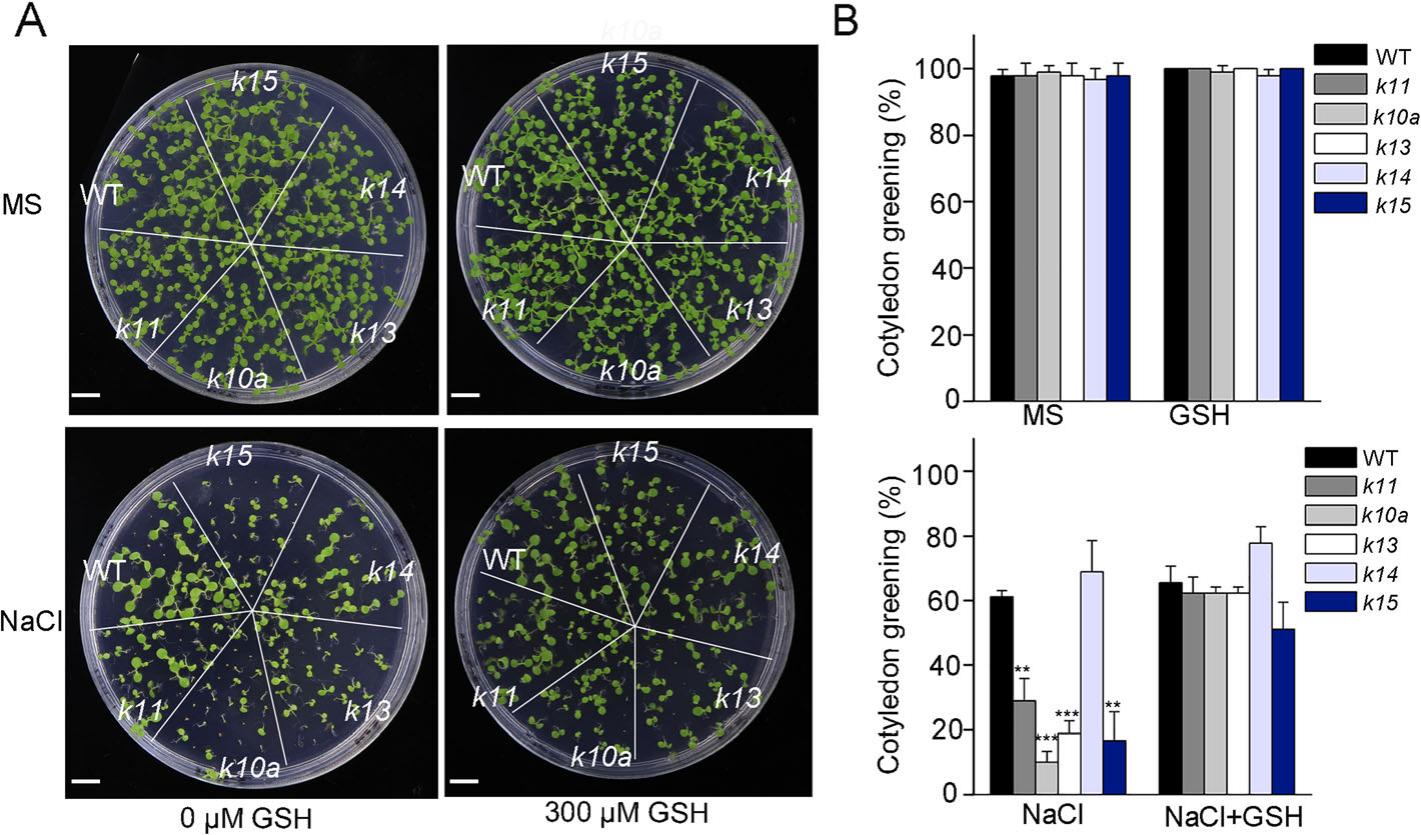
Mitochondrial ABC1K protein participate in salt stress response. The cotyledon greening phenotype of the *abc1k* mutants in response to NaCl treatment and the recovery of this phenotype by GSH. The seeds of the *abc1k* mutants were planted on MS medium without or with 200 mM NaCl for 10 days. The recovery of the cotyledon greening phenotype was test with 100 μM GSH (**a**). Bars = 5 mm. The rates of cotyledon greening (**b**). Error bars indicate means ± SD (*n* = 100). Asterisks indicate significant difference with the corresponding wild type (**P < 0.01, ***P < 0.001, *t*-test)

We also examined the possible role of other mitochondrial localized ABC1Ks in salt stress tolerance. There are no differences between *k11, k13, k14* and *k15* mutants and wild type when grown on MS medium, however, the growth phenotypes of *k11*, *k13* and *k15* mutants were severely altered under 100 mM NaCl treatment (Fig. 7a, b; Additional file 4: S4). Since these mutants accumulated higher levels of ROS when exposed to salt stress (Fig. 1b, c), we determined whether the addition of the ROS scavenger GSH could revert the effects of salt stress. Consistent with our previous results using *10a* mutants, the salt hypersensitive phenotypes of these mutants were restored by the addition of GSH (Fig. 7a, b). Under NaCl treatment, the percentages of green cotyledons of *k11*, *k10a*, *k13* and *k15* were about 47, 16, 30 and 27% of that of the wild type, while the ratios were increased to 95, 95, 95 and 78%, respectively, when GSH was exogenously applied (Fig. 7b). These results demonstrate that the *Arabidopsis* mitochondria-localized ABC1K proteins play prominent roles in plant salt tolerance through maintaining ROS homeostasis.

## Discussion

As an atypical protein kinase, the yeast ancestral member ABC1K was reported to be required for coenzyme Q biosynthesis and thus mitochondrial oxidation metabolism [33]. In higher plants, the *abc1k* mutants were found to be highly sensitive to oxidative stress caused by high intensity light, Cd^2+^ or H_2_O_2_ [23–25]. In this study, we found that the T-DNA insertion mutants of the mitochondria ABC1K members are hypersensitive to salinity stress (Fig. 1b; Fig. 7). Detailed phenotypic characterization of two null mutants of *10a*, a member of the ancestral clade, revealed markedly stunted de-etiolation process and growth inhibition under salinity stress (Fig. 3c, d. e). In consistent with the dramatic response phenotype, the expression of the salt-inducible marker genes was also significantly higher in the *10a* mutants than in wild type (Fig. 3f), suggesting a sensitive-response of the mutants to salt stress. In contrast to mature plants, seedlings during de-etiolation period are more sensitive to various abiotic stresses like drought, salinity, light and temperature stress [34, 35]. The *10a* mutants show a strong defect in cotyledon greening, which may be associated with its relatively high expression in siliques and cotyledons (Fig. 2a, b; Fig. 3c). This also indicates that ABC1K10a may play a prominent role in early seedling development under salinity stress. Our loss-of-function and gain-of-function analyses revealed that ABC1K10a is a positive regulator of salinity tolerance in *Arabidopsis* (Fig. 3c; Additional file 1: S1).

Abiotic stresses often result in the accumulation of cellular ROS and thus cause oxidative stress as a secondary stress. Although ROS have been recognized as signaling molecules, excess ROS induced by stress conditions damage cellular components such as DNA, RNA, and proteins [36]. Mitochondria are a major source of ROS and play an essential role in regulating important cellular metabolic processes [37]. Our results suggest that the mitochondrial ABC1K10a play a prominent role in mitochondria function under salt stress condition (Fig. 6a, b), and defective of this gene results in overaccumulation of ROS (Fig. 5e, f), which is likely originated from mitochondria. The salt hypersensitive phenotype of *10a* mutants can be rescued by GSH (Fig. 5c, d), a non-enzymatic antioxidant that counteracts ROS accumulation [15], indicating that the salt hypersensitivity of *10a* mutants is caused by overaccumulation of mitochondrial originated ROS that result in oxidative stress damage. ROS has also been reported to regulate various transcriptional regulation, for instance, when *Arabidopsis thaliana* seedlings were exposed to H_2_O_2_, 459 genes expression are enhanced more than 2-fold [38]. Previous research indicated that the deficiency of ROS scavengers catalase caused redundant ROS accumulation and diverse genes induced expression [39]. The similar proofs were observed in *abc1k10a* mutants, more ROS accumulated, while enhanced genes expression of *RD29A*, *KIN1* and *COR15b*, which may be triggered by endogenous ROS accumulation (Fig. 3f).

Plant cellular respiration incorporates COX pathway and AP pathway. When COX pathway is inhibited under stress conditions, AOX functions in preventing UQ over-reduction, alleviating ROS production, and maintaining cellular respiration status [39]. In the *10a* mutants, the expression of *AOX* gene was reduced under salt stress, suggesting that the AP pathway was also restrained, which resulted in enhanced ROS production in mitochondria and oxidative damage in the mutants. Mitochondria are important for the maintenance of cellular redox equilibrium and the mitochondrial complex III has been proved to be a major site for ROS production [9]. The *10a* mutants showed diminished mitochondrial complex III, suggesting that *K10a* may modulate the accumulation of mitochondrial complex III to fine-tune ROS level in response to salinity stress (Fig. 6b).

ABC1K proteins share conserved motifs including the catalytic loop, NTP-binding pockets, and Mg^2+^ chelation site, which suggests similar functions of these proteins in plants [21, 40]. Our study indicates that, in addition to the *10a* mutants, several other *abc1k* mutants also show salt hypersensitive phenotype and overaccumulation of ROS under salt stress (Fig. 1b; Fig. 7), revealing a conservative role of ABC1K family proteins in plant response to salinity stress. Meanwhile, the plastid clade members ABC1K1, ABC1K3 and ABC1K8 have also been shown to play important roles in oxidative stress response [25, 26]. Therefore, the ABC1K family proteins might be evolutionarily conserved proteins functioning in modulating cellular ROS production in response to abiotic stresses in plants.

## Conclusions

Here, we found the mitochondria-localized ABC1K10a functions in plant salt stress tolerance by regulating ROS accumulation. Our data suggest that *ABC1K10a* expression is induced by salt treatment, and the mutation of this gene results in significant sensitive to salt stress and severe oxidative stress. Further studies reveal that the defects of mitochondria electron transport chain are responsible for ROS overaccumulation in the *10a* mutants. Other ABC1K proteins in mitochondria also regulate ROS production and salt stress tolerance.

## Methods

### Plasmid construction

The coding sequence of *K10a* was inserted into *pCAMBIA1302* to create a YFP tagged 10a protein at the C terminal. The *K10a* coding sequence was also cloned into the *pDONR™221* vector by using the Gateway BP Clonase™ II Enzyme Mix (Invitrogen). And through LR reaction (Invitrogen) the CDS sequence was cloned into the binary vector *pYL436* to generate a 10a protein with nine Myc-tags at its C-terminal [41].

The genomic sequence including 1500 bp upstream of *K10a* transcription start codon and the entire coding sequence of *K10a* without stop codon was amplified and cloned into *pCAMBIA1300* to generate *proABC1K10a::ABC1K*1*0a-GFP* vector. The promoter used in the vector *proABC1K10a::ABC1K10a-GFP* was the same as in *proABC1K10a::GUS*. Primers used for vector constructs have been listed in the Additional file 5: Table S1.

### Plant materials and growth conditions

The wild type in this study refers to *Arabidopsis thaliana* var. Columbia-0. The *abc1k10a-1* (SALK_091799), *abc1k10a-2* (SALK_067438), *abc1k11*(SALK_009686C), *abc1k13* (SALK_065389), *abc1k14* (SALK_080026) and *abc1k15*(SALK_056597) mutants were obtained from Nottingham Arabidopsis Stock Centre (NASC, *http://arabidopsis.info*). The locations of the T-DNA insertions were at the promoter and the second exon of the AT1G11390 gene, respectively. The homozygotes were identified using the left/right genomic primer (LP and RP) and LBa1 of *pBIN-pROK2*. *Arabidopsis thaliana* were grown in a greenhouse with appropriate growth conditions as follows: 100 μM photons m^− 2^ s^− 1^, 23 °C/21 °C (day/night), 16-h-light/8-h-dark cycle and 55% relative air humidity. *Nicotiana benthamiana* plants were grown at 30 °C under 16-h-light/8-h-dark cycle. Primers used for homozygote identification have been listed in the Additional file 5: Table S1.

The transgenic plants were generated by using the *Agrobacterium tumefaciens*-mediated floral dip method [42, 43]. The constructs of *35S::ABC1K10a-YFP/Myc* and *proABC1K10a::ABC1K10a-YFP* were introduced into wild type and the *10a* mutant, producing transgenic lines for K10a overexpression and mutant complementation, respectively. Transgenic seeds were screened on 1/2 MS medium with appropriate antibiotics, and the T3 plants were used for relevant experiments.

### Abiotic stress treatments

For the cotyledon greening assay, seeds harvested from the same batch were sterilized for 10 min with 10% (v/v) sodium hypochlorite, rinsed five times with sterile-deionized water, and then grown on 1/2 MS medium with different concentrations of NaCl or GSH. The seeds were kept at 4 °C for 2 days, and then incubated at 23 °C under long-day photoperiod (16-h-light/8-h-dark) illumination for 8–10 days before photographed and statistics analyzed described [44].

For salt stress treatment analysis, 5-day-old seedlings grown on normal medium were immersed in 1/2 MS liquid medium with or without 200 mM NaCl for the indicated times [45, 46]. The plants were collected, frozen in liquid nitrogen and preserved at − 80 °C for further use.

### GUS staining assay

Histochemical analysis of promoter-GUS was performed as described [30]. 5-day-old transgenic seedlings of *proABC1K10a::GUS* grown on 1/2 MS medium were soaked in 1/2 MS liquid medium with 200 mM NaCl for 3 or 6 h and then used for GUS staining. Different organs of mature plants were collected for GUS staining. The samples were submerged in cold 90% acetone for 20 min, washed with rinse solution (50 mM phosphate buffer, pH 7.2, 0.5 mM K-ferrocyanide, 0.5 mM K-ferricyanide), and then stained in the pre-prepared staining solution (rinse solution containing 2 mM X-Gluc) in the dark for a consistent incubation time. After removing the staining solution, samples were washed with 15, 35, 50, 70% ethanol for 15 min each, and then 100% ethanol for 10 min to remove the chlorophyll. The seedlings and organs were photographed with an Olympus MVX10 stereomicroscope.

### Confocal microscopy

To determine the subcellular localization of K10a, the vector *pro35S:*:*ABC1K10a-YFP* was transformed into *Agrobacterium strain GV3101* [43, 44]. After 12 to 16 h culturing, the *Agrobacteria* cells were collected, resuspended to the transformation buffer with OD600 ≈ 1.0, and injected into *Nicotiana benthamiana* young leaves. After 48 h co-culturing, the infiltrated leaves were stained with Mito-Tracker orange as a mitochondrial fluorescence marker. Fluorescent signals were observed with a Carl Zeiss LSM710 META laser scanning microscope.

### Quantitative RT-PCR

For qRT-PCR, 5-d-old wild type and *10a* mutant seedlings were treated with 1/2 MS liquid medium containing 200 mM NaCl for 3 or 6 h. Total RNAs were extracted using TRIzol reagent (Invitrogen), treated with RNase-free DNase set (Qiagen), and reverse transcribed with M-MLV reverse transcriptase (Promega). For real-time PCR assays, reactions were performed with ChamQ Universal SYBR qPCR Master Mix (Vazyme) using three independent biological replicates. The relative real-time PCR amplification was described, with *ACTIN 2/8* as the internal control [47]. Primers used for qRT-PCR have been listed in the Additional file 5: Table S1.

### Protein gel blotting

For detecting the protein levels of K10a-GFP, 5-day-old transgenic seedlings were treated with 1/2 MS liquid medium containing 200 mM NaCl at the indicated times, and then ground into fine powder in liquid nitrogen. The proteins were extracted in extraction buffer (50 mM Tris-HCl, pH 7.4, 150 mM NaCl, 5 mM EDTA, 1 mM PMSF, 5 mM DTT, 0.5% SDS) and quantified with BCA Protein Assay Kit (TaKaRa) as described [48]. Equal amount of proteins for each sample was separated with 12% SDS-PAGE gel and transferred onto a nitrocellulose membrane. The membrane was blocked with 5% non-fat milk, incubated with polyclonal anti-GFP (1/2000) or anti-Actin (1/5000) antibodies. The signals were visualized with an X-ray film (Kodak).

### Measurement of ROS in plants

For the measurement of ROS, 5-day-old wild type and *abc1k* mutants were treated with 1/2 MS medium containing 200 mM NaCl at the indicated times and stained with the dyes. The ROS levels were analyzed as previously described [49].

During NBT staining, the seedlings were incubated in reaction buffers for 15 mins which were composed by 20 mM K-phosphate buffer (pH 6.2), 0.1 M NaCl and 1 mM NBT. The reaction was performed in the dark in order to inhibit the decomposition of NBT. The seedlings were washed with water for three times to remove excess dyes, then incubated in acidified buffer (10 mL of methanol, 2 mL of HCl, 38 mL of water) and basic buffer (7% NaOH in 60% ethanol) for 15 min, respectively. The mazarine can be observed, and the pigments were removed by with gradient concentration of ethanol treatment. The cotyledons or roots were photographed using Olympus MVX10 stereomicroscope.

For DCFH-DA staining, the seedlings were incubated in reaction buffer (20 mM K-phosphate, pH 6.0, 50 mM DCFH-DA) in darkness for 10 min. The cotyledons were photographed using Carl Zeiss LSM710 META laser scanning microscope at 488 nm.

### Blue native-PAGE and complex activity assay

To detect the accumulation of mitochondrial complexes, 5-day-old wild type and the *10a-1*, *10a-2* mutants grown on MS medium or MS medium containing 100 mM NaCl were used for the preparation of mitochondria membrane proteins as previously described [30, 50, 51]. For the analysis of mitochondrial protein abundance, 50 μg mitochondria proteins from each sample were separated by a 4.5 to 16% gradient Blue-Native PAGE (Invitrogen, BN1002BOX), stained with coomassie brilliant blue (CBB), faded with 10% methanol and 10% acetic acid (v/v) and then imaged.

### Bioinformatics and phylogenetic analysis

*Arabidopsis* ABC1Ks protein sequences obtained from the UniProt (*https://www.uniprot.org/*) were used for homologous alignment. Sequence alignment and the drawing of evolutionary trees were carried out by using the software MEGA4.

### Quantification and statistical analysis

Quantitative data is expressed as mean ± SD and mean ± SE. Statistical significance among various treatments and materials was performed by one-way ANOVA and two-way ANOVA. Statistical significance was set at * *P* < 0.05, ** *P* < 0.01, *** *P* < 0.001.

### Availability of data and materials

All data generated or analysed during this study are included in this published article and its supplementary information files. The plant materials during the current study available from the corresponding author on reasonable request.

## Supplementary information

**Additional file 1: S1** The *ABC1K10a* overexpression lines are more tolerant to salt stress. (A) Seedlings from different genotypes, wild type, *10a-1* mutant, *10a-2* mutant and two independent overexpressing lines (OE-1 and OE-2) were grown on MS medium or MS medium supplemented with 100 mM NaCl. Bar = 500 μm. (B) Quantification of cotyledon greening. Three biological replicates were conducted. Error bars indicate ± SD (*n* = 100). Asterisks indicate significant differences with the corresponding wild type (**P < 0.01, *P < 0.05, *t*-test).

**Additional file 2: S2** Expression of ABC1K10a is induced by NaCl treatment at translational level. To extract the total proteins, 5-day-old transgenic seedlings expressing *ABC1K10apro::ABC1K10a-GFP* were treated with 200 mM NaCl for 0, 3 or 6 h. Protein extracts were analyzed by western blotting with α-GFP antibody. Actin was used as a control.

**Additional file 3: S3** The abundance of the mitochondrial complexes in wild type and the *10a* mutants.

**Additional file 4: S4** Molecular characterization of *ABC1K* family members. (A) Locations of the T-DNA insertion alleles. (B) Expression of *ABC1Ks* detected by qRT-PCR.

**Additional file 5: Table S1** Primers used in this study.

## Data Availability

Not applicable.

## Abbreviations

ABC1K: Activity of bc1 complex kinase
ROS: Reactive oxygen species
SOS: Salt overly sensitive
SCaBP8: SOS3-like calcium binding protein 8
PKS5: Protein Kinase SOS2-Like 5
H_2_O_2_: Hydrogen peroxide
^1^O_2_: Singlet oxygen
OH: Hydroxyl radical
O_2_˙ˉ: Superoxide anion radical
UQ: Ubiquinone
NO: Nitric oxide
AtMT2b: A type 2 metallothionein
AtVDAC3: Voltage-dependent anion channel 3
MMP: Mitochondrial membrane potential
GSH: Tripeptide glutathione
SOD: Superoxide dismutase
CAT: Catalase
APX: Ascorbate peroxidase
GPX: Glutathione peroxidase
NBT: Nitroblue tetrazolium
RD29A: Responsive to desiccation 29A
KIN1: Kinase 1
COR15b: Cold regulated 15b
H_2_DCF-DA: 2′7’-dichlorofluorescin diacetate
COX: Cytochrome c oxidase
AP: Alternative pathway
AOX: Alternative oxidase
