# Supplementary figures and images for: ABC1K10a, an atypical kinase, functions in plant salt stress tolerance
Source: BMC Plant Biol. 2020 Jun 10;20:270. doi: 10.1186/s12870-020-02467-4 (PMC7288548; doi:10.1186/s12870-020-02467-4)

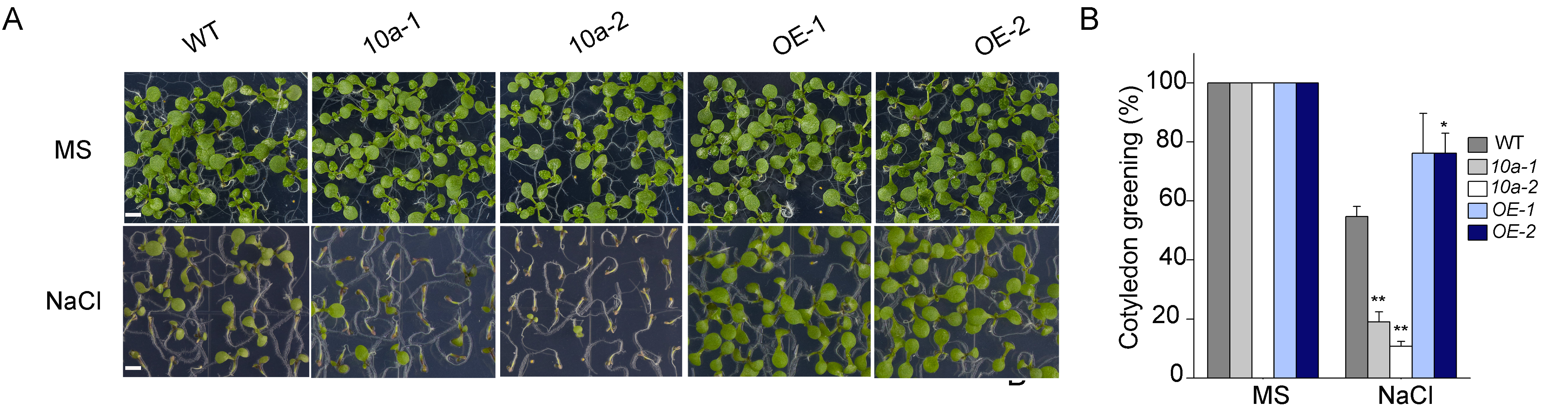

Supplement: Supplementary file 1 — Additional file 1: S1 The ABC1K10a overexpression lines are more tolerant to salt stress. (A) Seedlings from different genotypes, wild type, 10a-1 mutant, 10a-2 mutant and two independent overexpressing lines (OE-1 and OE-2) were grown on MS medium or MS medium supplemented with 100 mM NaCl. Bar = 500 μm. (B) Quantification of cotyledon greening. Three biological replicates were conducted. Error bars indicate ± SD (n = 100). Asterisks indicate significant differences with the corresponding wild type (**P < 0.01, *P < 0.05, t-test). [file 12870_2020_2467_MOESM1_ESM.tif]

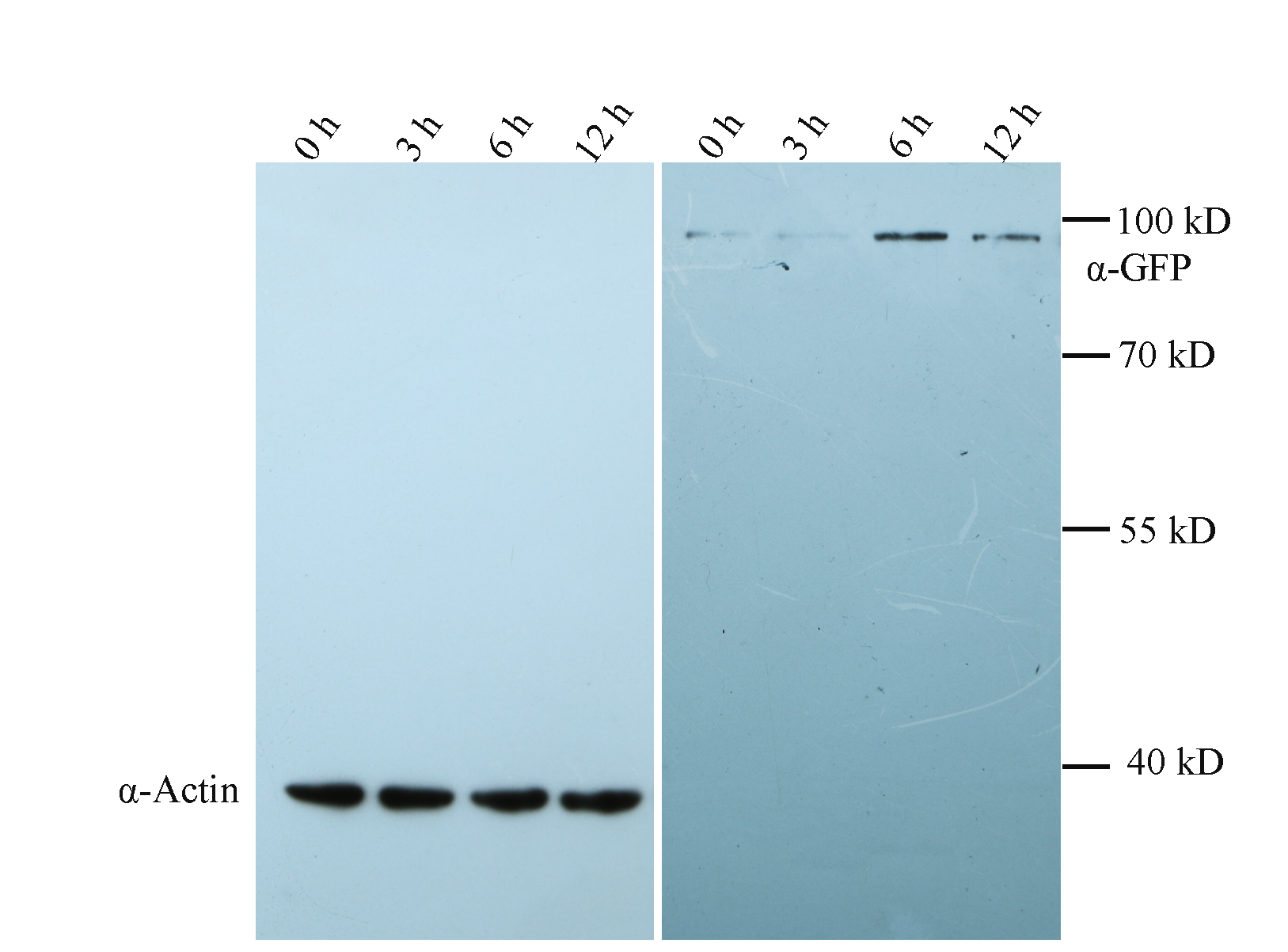

Supplement: Supplementary file 2 — Additional file 2: S2 Expression of ABC1K10a is induced by NaCl treatment at translational level. To extract the total proteins, 5-day-old transgenic seedlings expressing ABC1K10apro::ABC1K10a-GFP were treated with 200 mM NaCl for 0, 3 or 6 h. Protein extracts were analyzed by western blotting with α-GFP antibody. Actin was used as a control. [file 12870_2020_2467_MOESM2_ESM.tif]

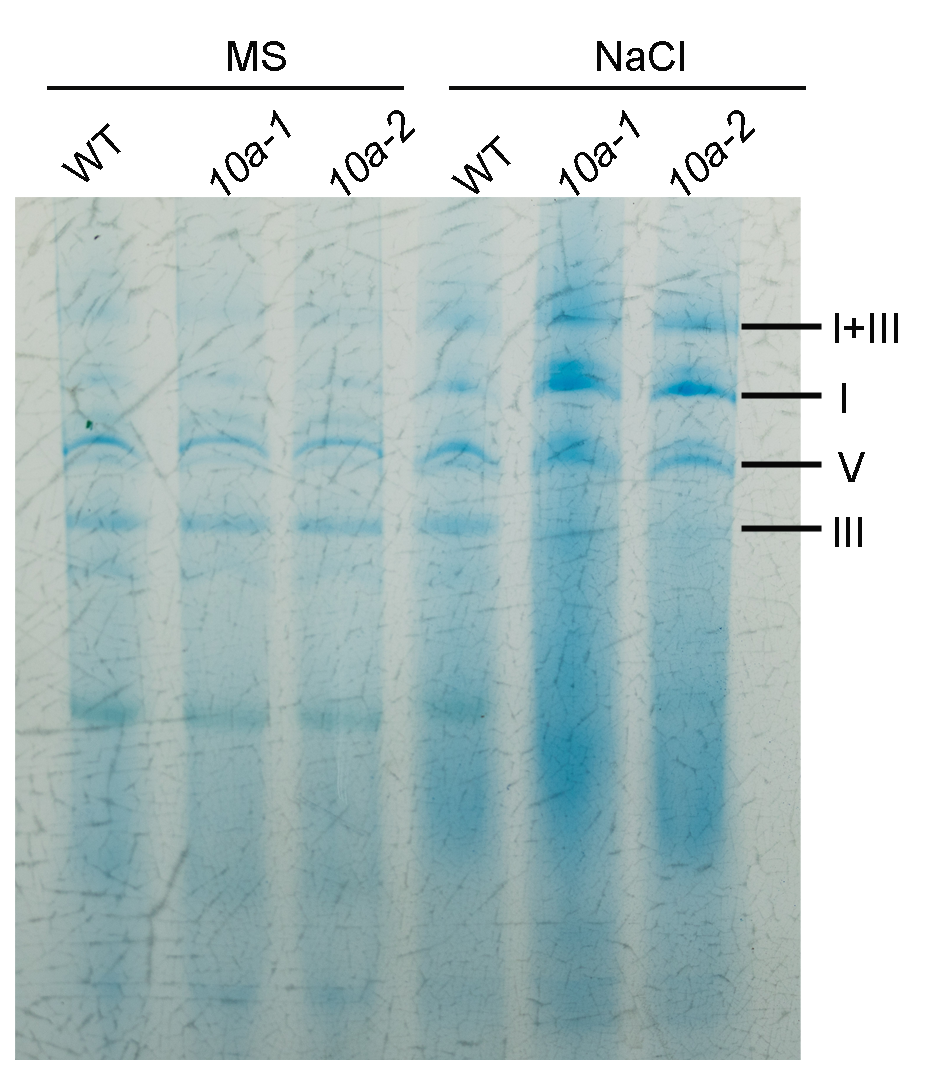

Supplement: Supplementary file 3 — Additional file 3: S3 The abundance of the mitochondrial complexes in wild type and the 10a mutants. [file 12870_2020_2467_MOESM3_ESM.tif]

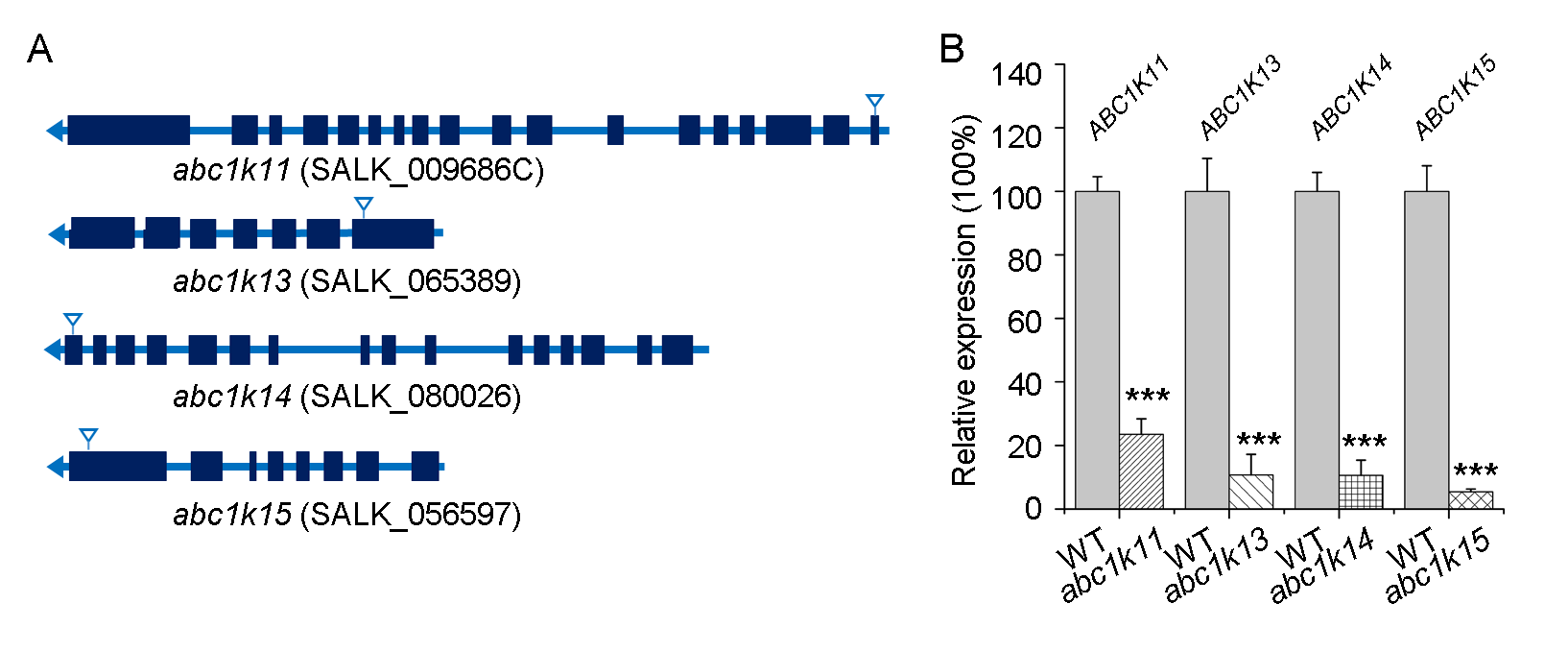

Supplement: Supplementary file 4 — Additional file 4: S4 Molecular characterization of ABC1K family members. (A) Locations of the T-DNA insertion alleles. (B) Expression of ABC1Ks detected by qRT-PCR. [file 12870_2020_2467_MOESM4_ESM.tif]
