# Supplementary material for: ABC1K10a, an atypical kinase, functions in plant salt stress tolerance
Source: BMC Plant Biol. 2020 Jun 10;20:270. doi: 10.1186/s12870-020-02467-4 (PMC7288548; doi:10.1186/s12870-020-02467-4)
Supplement: Supplementary file 5 — Additional file 5: Table S1 Primers used in this study. [file 12870_2020_2467_MOESM5_ESM.docx]

Table S1 Primers used in this study.

| **Gene name** | **Primers** |
| --- | --- |
| **Primers for**  **qRT-PCR** | |
| *ABC1K10a*-F | TTAAGTAATCATCTGTCTCG |
| *ABC1K10a*-R | TACTAATACGCTCAGAATAC |
| *KIN1*-F | TGGAGCTGGAGCACAACA |
| *KIN1*-R | GACCCGAATCGCTACTTGTTC |
| *COR15B*-F | AAAGCAGAGTGGTGTTGGTACCGT |
| *COR15B*-R | TCATCGAGGATGTTGCCGTCACTT |
| *RD29A*-F | GCCGAGAAACTTCAGATTGG |
| *RD29A*-R | CCATTCCTCCTCCTCCTTTC |
| *ABC1K11*-F | AGATCATCAAGAGAGGAAGC |
| *ABC1K11*-R | TCCAGCACCCATGAAAGCAT |
| *ABC1K13*-F | CCGATTCCTCTTTTTCGCTTTC |
| *ABC1K13*-R | ACCGGGTTCTGCAAAAGGTA |
| *ABC1K14*-F | CTTCCGAGACTCACCTTCCA |
| *ABC1K14*-R | AAGTATACTTGTAATCAGCG |
| *ABC1K15*-F | AAAGCTTGGGATTCAGCCCT |
| *ABC1K15*-R | TCTGCAAATGTTTGACTCAC |
| *ACT2/8*-F | TAACAGGGAGAAGATGACTCAGATCA |
| *ACT2/8*-R | AAGATCAAGACGAAGGATAGCATGAG |
| **Primers for homozygote identification** | |
| *abc1k10a-1*-LP | ATGGGAAACAAAAATCCGATC |
| *abc1k 10a-1*-RP | TGCAATACAAAGTCCCAGCTC |
| *abc1k 10a-2*-LP | GATAAAAGCCTCTGGATTGGG |
| *abc1k10a-2*-RP | ATTCCCCAAGGATTTGTGTTC |
| *abc1k11*-LP | AATGCCAATCTTGCTTTCATC |
| *abc1k11*-RP | TACGTCGAAAACCAACCTCAG |
| *abc1k13*-LP | GTAGAAAACAGCATTGCCTGC |
| *abc1k13*-RP | CTCAGGACTTTCGAATCAACG |
| *abc1k14*-LP | ACAATGCACAGTTTGGGTTTC |
| *abc1k14*-RP | TTCAATCAAAATGAATTCTTGTCC |
| *abc1k15*-LP | ATCAGCTCACCAAAAACATGC |
| *abc1k15*-RP | AGCTCTCTCACTGCTTCCCTC |
| **Primers for vector constructs** | |
| K10a-OE-YFP-L | GGGGTACCATGTCAAGAGTTTTGATCTCT |
| K10a-OE-YFP-R | GCTCTAGACGGCGCCATCAGACCATCCA |
| K10a-COM-YFP-L | CCGAATTCCTGTTTTCTGTATCAAATTTACAG |
| K10a-COM-YFP-R | GCTCTAGACGGCGCCATCAGACCATCCA |
| K10a-GUS-L | GCGAATTCCTGTTTTCTGTATCAAATTTACAG |
| K10a-GUS-R | GCAAGCTTAGCGACGAAGATGGTGAGAAACC |
| K10a-Myc-L | GGGGACAAGTTTGTACAAAAAAGCAGGCTtcATGTCAAGAGTTTTGATCTCT |
| K10a-Myc-R | GGGGACCACTTTGTACAAGAAAGCTGGGTCCGGCGCCATCAGACCATCCA |
